# Supplementary material for: Genome Analysis of the G6P6 Genotype of Porcine Group C Rotavirus in China
Source: Animals (Basel). 2022 Oct 27;12(21):2951. doi: 10.3390/ani12212951 (PMC9657714; doi:10.3390/ani12212951)
Supplement: Supplementary file 1 [file animals-12-02951-s001.zip › animals-1997300-Table S1.pdf]

Table S1. Primers used in this study.

Primer	Sequence(5′-3′)	Product(bp)
F-RVC-VP1	TAAAAAAAATGGCGCAGTCC	
R-RVC-VP1	AGCCACAATATGCAGTCC	3306
F-RVC-VP2	CAAATCGTCCAAGATGATAAGC	
R-RVC-VP2	CAGAATTTGAGGTCATC	2713
F-RVC-VP3	AAAAAAGCCCAACACGCAAT	
R-RVC-VP3	AGCCACATCATACGGTAGAGG	2162
F-RVC-VP4	GGATCAATGGCGTCCTCACTTT	
R-RVC-VP4	CATAATAAGTCGATCTCCTCAC	2248
F-RVC-VP6	GCATTTAAAATCTCATTCACA	
R-RVC-VP6	AGCCACATAGTTCACATTTCA	1352
F-RVC-VP7	AGCTGTCTGACAAACTGGT	
R-RVC-VP7	AGCCACATGATCTTGTTTAC	1045
F-RVC-NSP1	AAGAGCTAGGCTTGGAGGTGT	
R-RVC-NSP1	GCCACATAGAGTTTAGTC	1256
F-RVC-NSP2	ATGGCCGAGCTAGCCTGTTTC	
R-RVC-NSP2	TTCCTGTTGCTTGTAACCTTC	973
F-RVC-NSP3	TTGCTGGAAATGGCGACTCA	
R-RVC-NSP3	CTGCCATTGACCTTTTACTT	1315
F-RVC-NSP4	AAATCGCTTTGCTCTACG	
R-RVC-NSP4	GCTAGAAATGGACAGAATGG	527
F-RVC-NSP5	ACAATGTCCGATTTCGGAAT	
R-RVC-NSP5	TCAATGCCGATTCCAGTCA	687
F-SADS-N	CAGGTCTTGGTGTTCGCAATCG	
R-SADS-N	ACCGTGCTGAACGAGGTCACT	449
F-TGEV-S	AAGGAAGGGTAAGTTGCTCA	
R-TGEV-S	GGTCCATCAGTTACGCCGAA	1255
F-PORV-VP7	GGCTTTAAAAGAGAGAATTTC	

R-PORV-VP7	GGTCACATCATACAGTTCTAAC	1062
F-PDCoV-S	GCAACGCCTATTATCTCG	
R-PDCoV-S	ACATGCCATTGTTTACTG	1205
