# Supplementary material for: Genome Analysis of the G6P6 Genotype of Porcine Group C Rotavirus in China
Source: Animals (Basel). 2022 Oct 27;12(21):2951. doi: 10.3390/ani12212951 (PMC9657714; doi:10.3390/ani12212951)
Supplement: Supplementary file 1 [file animals-12-02951-s001.zip › animals-1997300-Table S2.pdf]

Supplementary Table S2. Reference sequence used in this study.

The RVC VP1 genes used in this study.

Strain	Host	Country	Collection date	Accession No.
Y/03	Bos taurus	Japan:Yamagata	2003-12-17	AB874606
Y/2/04	Bos taurus	Japan:Yamagata	2004-01-13	AB874608
Y/08	Bos taurus	Japan:Yamagata	2008-04-08	AB874610
RVC/Human-wt/JPN/HI-49/2006/G4P2	Homo sapiens	Japan	2006	LC129066
RVC/Human-wt/JPN/HO-63/2005/G4P2	Homo sapiens	Japan	2005	LC129069
RVC/Human-wt/JPN/OS-270/2012/G4P2	Homo sapiens	Japan	2012	LC129072
RVC/Human-wt/IND/NIV8831/Solapur2010/G4P2	Homo sapiens	India	2010	MG709364
RVC/Human-wt/IND/NIV22208/Solapur2011/G4P2	Homo sapiens	India	2010	MG709365
RVC/Human-wt/IND/NIV1418974/Miraj2014/G4P2	Homo sapiens	India	2014	MG709366
RVC/Dog-wt/HUN/174/2012/G10P8	dog	Hungary	2012	KP988013
ROTVP1A				M74216
RVC/Pig-wt/Tochigi-2/2014/G6P4	Sus scrofa	Japan: Tochigi	2014-04-25	LC122602
RVC/Pig-wt/Ishi-1/2015/G13P4	Sus scrofa	Japan: Ishikawa	2015-10-23	LC122624
CJ3-6	Sus scrofa	Japan:Kagoshima	2002-05	LC307078
86-K5	Sus scrofa	Japan:Chiba	2008-07	LC307090
87-I4	Sus scrofa	Japan:Ibaraki	2008-09	LC307091
RVC/Pig-wt/USA/RV0143/2011/G6P5	Pig	USA	Dec-2011	MN809633
RV0104	Pig	USA	22-Jul-2011	MN809644
RVC/Pig-wt/BEL/12R021/2012/G3P5_VP1	Sus scrofa	Belgium	2012	KP982879
RVC/Pig-wt/CAN/A11-154/2014/G6P5	pig	Canada	2014	KY909996
RVC/Pig-wt/USA/NC38/2012/G6P5	pig	USA:NC	14-Mar-2012	MG451440
RVC/Pig-wt/ESP/P1C/2017/GXP4	Sus scrofa	Spain	01-Nov-2017	MK953072
RVC/Pig-wt/ESP/VC20B/2017/G6P5	Sus scrofa	Spain	16-Mar-2017	MK953078
PoRVC_VP1_VIRES_HeB02_C2	Sus scrofa	China	Apr-2017	MK379289
RVC/CHN/JS02/2018/G6P6	Sus scrofa	China	2018	ON068577

The RVC VP2 genes used in this study.

**Strain**	**Host**	**Country**	**Collection date**	**Accession No.**
Cowden	swine	USA	1980	FJ970917
RVC/Pig-wt/Tochigi-1-1/2015/G9P4	Sus scrofa	Japan: Tochigi	2015-11-27	LC122593
RVC/Pig-wt/Tochigi-2/2014/G6P4	Sus scrofa	Japan: Tochigi	2014-04-25	LC122603
RVC/Pig-wt/Tottori-KT01/2015/G13P4	Sus scrofa	Japan: Tottori	2015-07-15	LC122614
RVC/Pig-wt/Ishi-1/2015/G13P4	Sus scrofa	Japan: Ishikawa	2015-10-23	LC122625
CJ3-6	Sus scrofa	Japan:Kagoshima	2002-05	LC307099
CJ59-32	Sus scrofa	Japan:Iwate	2003-10	LC307108
86-H3	Sus scrofa	Japan:Chiba	2008-07	LC307109
86-H5	Sus scrofa	Japan:Chiba	2008-07	LC307110
91-G10	Sus scrofa	Japan:Chiba	2008-10	LC307113
91-G7	Sus scrofa	Japan:Chiba	2008-10	LC307114
91-H5	Sus scrofa	Japan:Chiba	2008-10	LC307115
134-9	Sus scrofa	Japan:Miyazaki	2010-03	LC307118
RVC/Pig-wt/USA/OK.5.68/2008	Sus scrofa	USA	02-Jan-2008	MH282886
PoRVC_VP2_VIRES_GZ04_C1	Sus scrofa	China	Apr-2017	MK379221
PoRVC_VP2_VIRES_HeB02_C1	Sus scrofa	China	Apr-2017	MK379291
PoRVC_VP2_VIRES_NM02_C1	Sus scrofa	China	Apr-2017	MK379390
PoRVC_VP2_VIRES_NX02_C1	Sus scrofa	China	Apr-2017	MK379467
RVC/Dog-wt/HUN/174/2012/G10P8	dog	Hungary	2012	KP988014
RVC/Human-wt/ITA/PR2593/2004/G4P2	Homo sapiens	Italy	2004	KT206186
RVC/Human-wt/ITA/PR713/2012/G4P2	Homo sapiens	Italy	2012	KT206197
RVC/SJZ217/CHN/2015	Homo sapiens	China	Apr-2015	KY865345
Y/08	Bos taurus	Japan:Yamagata	2008-04-08	AB874616
Shintoku	Bos taurus	Japan		AB738406
Y/1/04	Bos taurus	Japan:Yamagata	2004-01-05	AB874613
RVC/Pig-wt/BEL/12R021/2012/G3P5_VP2	Sus scrofa	Belgium	2012	KP982880
RVC/CHN/JS02/2018/G6P6	Sus scrofa	China	2018	ON068578

The VP3 genes used in this study.

Strain	Host	Country	Collection date	Accession No.
RVC/Pig-wt/Tochigi-1-1/2015/G9P4	Sus scrofa	Japan: Tochigi	2015-11-27	LC122594
RVC/Pig-wt/Ishi-1/2015/G13P4	Sus scrofa	Japan: Ishikawa	2015-10-23	LC122626
CJ32-3	Sus scrofa	Japan:Akita	2002-10	LC307181
86-K5	Sus scrofa	Japan:Chiba	2008-07	LC307186
CJ33-5	Sus scrofa	Japan:Aomori	2002-11	LC307182
CJ59-32	Sus scrofa	Japan:Iwate	2003-10	LC307184
87-I4	Sus scrofa	Japan:Ibaraki	2008-09	LC307187
RVC/Pig-wt/Tochigi-2/2014/G6P4	Sus scrofa	Japan: Tochigi	2014-04-25	LC122604
CJ27-1	Sus scrofa	Japan:Aomori	2002-09	LC307179
86-H3	Pig	Japan:Chiba	2008-07	LC307185
CJ10-1	Sus scrofa	Japan:Kagoshima	2002-05	LC307176
Cowden	Pig	USA	1991	M74219
CAU 10-312	Homo sapiens	South Korea	2010	HQ896712
BS347	Homo sapiens	Bangladesh	2005	HQ185634
V508	Homo sapiens	India	2001	HQ185631
Y/08	Bos taurus	Japan:Yamagata	2008-04-08	AB874622
Y/10	Bos taurus	Japan:Yamagata	2010-11-04	AB874623
Toyama	Bos taurus	Japan	2010	AB738414
RVC/Dog-wt/HUN/174/2012/G10P8	dog	Hungary	2012	KP988015
RVC/CHN/JS02/2018/G6P6	Porcine	China	2018	ON068579

The RVC VP4 genes used in this study.

**Strain**	**Host**	**Country**	**Collection date**	**Accession No.**
RVC/Human-wt/HUN/ERN6216/2013/G4P2	Homo sapiens	Hungary	2013	KP776602
RVC/Human-wt/RUS/Omsk08-386/2008	Homo sapiens	Russia: Omsk	Apr-2008	JN934896
RVC/Human-wt/JPN/HO-61/2005/G4P2	Homo sapiens	Japan	2005	LC129051
RVC/SJZ217/CHN/2015	Homo sapiens	China	Apr-2015	KY865347
RVC/Pig-wt/CZE/P8/2011	Pig	Czech Republic	Nov-2010	KM099261
RVC/Pig-wt/CZE/P9/2011	Pig	Czech Republic	Nov-2010	KM099262
RVC/Pig-wt/CZE/P303/2011	Pig	Czech Republic	Dec-2011	KM099263
RVC/Pig-wt/KOR/07-74-11/2007/G1Px	porcine	South Korea	2007	KJ814470
RVC/Pig-wt/KOR/08-148-2/2008/GxPx	porcine	South Korea	2008	KJ814471
RVC/Pig-wt/KOR/2478/2012/GxPx	porcine	South Korea	2012	KJ814474
RVC/Pig-wt/KOR/2885/2012/G7Px	porcine	South Korea	2012	KJ814475
RVC/Pig-wt/KOR/1027/2012/G7Px	porcine	South Korea	2012	KJ814476
RVC/Pig-wt/Tottori-KT01/2015/G13P4	Sus scrofa	Japan: Tochigi	2015-07-15	LC122611
RVC/Pig-wt/USA/OK.5.68/2008	Sus scrofa	USA	02-Jan-2008	MH282888
RVC/Pig-wt/VNM/12129_51/VP4_c1	Sus scrofa	Viet Nam	27-Mar-2012	KX362446
RVC/Pig-wt/VNM/14249_25/VP4	Sus scrofa	Viet Nam	03-Apr-2012	KX362484
RVC/Pig-wt/CAN/A10-108/2014/G6P4	Pig	Canada	2014	KY910036
RVC/Pig-wt/CAN/A11-152/2014/G6P5	Pig	Canada	2014	KY910038
VC/Pig-wt/USA/MN14/2012/G1P7	Pig	USA:MN	08-Feb-2012	MG451606
RVC/Pig-wt/USA/NC27/2012/G3P7	Pig	USA:NC	02-Mar-2012	MG451616
RVC/Pig-wt/USA/MN31/2012/G9P6	Pig	USA:MN	06-Mar-2012	MG451619
RVC/Pig-wt/MEX/SO34/2012/G9P5	Pig	Mexico:SO	07-Mar-2012	MG451622
RVC/Pig-wt/USA/IA46/2012/G9P7	Pig	USA:IA	10-Apr-2012	MG451630
RVC/Pig-wt/USA/AR53/2012/G3P7	Pig	USA:AR	19-Apr-2012	MG451632
RVC/Pig-wt/USA/MN56/2012/G9P1	Pig	USA:MN	03-May-2012	MG451635
RVC/Pig-wt/ESP/VC20B/2017/G6P5	Sus scrofa	Spain	16-Mar-2017	MK953045
RVC/Pig-wt/VNM/12129_51/VP4_c2	Sus scrofa	Viet Nam	27-Mar-2012	KX362447
RVC/Pig-wt/USA/RV0104/2011/G3P18	pig	USA	22-Jul-2011	MT181131
CJ49-4	Porcine	Japan:Aomori	2003-08	AB902239
86-K5	Porcine	Japan:Chiba	2008-07	AB905242
91-G7 P4	Porcine	Japan:Chiba	2008-10	AB905245
91-G10	Porcine	Japan:Chiba	2008-10	AB905246
105-4	Porcine	Japan:Gifu	2009-03	AB905250
CJ16-4	Porcine	Japan:Ehime	2002-06	AB905234
CJ31-6	Porcine	Japan:Kagoshima	2002-10	AB905236
PoRVC_VP4_VIRES_GZ04_C2	Porcine	China	Apr-2017	MK379223
PoRVC_VP4_VIRES_HeB02_C3	Porcine	China	Apr-2017	MK379295
RVC/Pig-tc/USA/Cowden/1991/G1P1				M74218
RVC/CHN/JS02/2018/G6P6	Porcine	China	2018	ON068580

The RVC VP6 genes used in this study.

Strain	Host	Country	Collection date	Accession No.
RVC/Pig-wt/KOR/07-74-11/2007/G1PX	porcine	South Korea	2007	KJ814480
RVC/Pig-wt/KOR/09-15-7/2009/G6PX	porcine	South Korea	2009	KJ814481
RVC/Pig-wt/KOR/09-15-9/2009/G3PX	porcine	South Korea	2009	KJ814482
RVC/Pig-wt/KOR/08-128-1/2008/GXPX	porcine	South Korea	2008	KJ814483
CA-2	Pig	South Korea	2009	GQ925781
BRA634/05-Po	piglet	Brazil	May-2005	JF810445
RVC/Pig-wt/USA/CO76/2012/G6P5	Pig	USA:CO	10-Apr-2012	MG451721
RVC/Pig-wt/USA/MO33/2012/G6P5	Pig	USA:MO	07-Mar-2012	MG451685
RVC/Pig-wt/USA/IA55/2012/G1P4	Pig	USA:IA	24-Apr-2012	MG451703
RVC/Pig-wt/USA/IL22/2012/G6P5	Pig	USA:IL	28-Feb-2012	MG451675
BRA499/04-Po	Pig	piglet	Oct-2004	JF810444
RVC/Pig-wt/USA/IL75/2012/G6P5	Pig	USA:IL	02-Apr-2012	MG451720
RVC/Pig-wt/USA/IA46-3/2012/G3PX	Pig	USA:IA	10-Apr-2012	MG451697
RVC/Pig-wt/USA/RV0104/2011	Pig	USA	22-Jul-2011	KC164674
RVC/Pig-wt/USA/RV0143/2011	Pig	USA	Dec-2011	KC164677
CUK-5	Pig	South Korea	2010	HQ833829
CUK-6	Pig	South Korea	2010	HQ323753
06-92-1	Pig	South Korea	2009	FJ494690
RVC/Pig-wt/KOR/06-52-1/2006/GXPX	porcine	South Korea	2006	KJ814477
RVC/Pig-wt/CZE/P51/2009	pig	Czech Republic	Apr-2009	KF036281
RVC/Pig-wt/CZE/P62/2009	pig	Czech Republic	Apr-2009	KF036282
CJ31-6	Sus scrofa	Japan:Kagoshima	2002-10	AB889516
RVC/Pig-wt/Tochigi-2/2014/G6P[4]	Sus scrofa	Japan: Tochigi	2014-04-25	LC122601
CJ10-1	Sus scrofa	Japan:Kagoshima	2002-05	AB889512
CJ13-6	Sus scrofa	Japan:Kagoshima	2002-05	AB889513
RVC/Pig-wt/CZE/P131/2010	Pig	Czech Republic	Sep-2010	KF036286
RVC/Pig-wt/Tochigi-1-1/2015/G9P[4]	Pig	Japan: Tochigi	2015-11-27	LC122591
RVC/Pig-wt/USA/NC24/2012/G9P4	Pig	USA:NC	01-Mar-2012	MG451677
PoRVC_VP6_VIRES_HeB02_C1	Sus scrofa	China	Apr-2017	MK379296
PoRVC_VP6_VIRES_NM01_C1	Sus scrofa	China	Apr-2017	MK379361
PoRVC_VP6_VIRES_NX01_C1	Sus scrofa	China	Apr-2017	MK379436
RVC/Pig-wt/CZE/P111/2010	pig	Czech Republic	Sep-2010	KF036285
RVC/Pig-wt/CZE/P9/2011	pig	Czech Republic	Nov-2010	KM099255
RVC/Pig-wt/CZE/P72/2009	pig	Czech Republic	Apr-2009	KF036283
RVC/Pig-wt/CZE/P207/2010	pig	Czech Republic	Oct-2010	KF036288
RVC/Pig-wt/CZE/P21/2013	pig	Czech Republic	Mar-2013	KM099257
RVC/Pig-wt/CZE/P44/2013	pig	Czech Republic	Mar-2013	KM099258
Cowden	Cow			M94157
RVC/Pig-wt/KOR/04-105-2/2004/GXPX	porcine	South Korea	2004	KJ814478
06-144-2	porcine	South Korea		FJ494691
Wu82	human	China		EF528570

BS347	Homo sapiens	Bangladesh	2005	HQ185636
Y/2/04	Bos taurus	Japan:Yamagata	2004-01-13	AB874632
Yamagata	Bovine	Japan		AB108680
RVC/Dog-wt/HUN/174/2012/G10P8	Dog	Hungary	2012	KP988017
Ferret Rota C-MSU	Ferret	USA	09-May-2003	KF578541
RVC/CHN/JS02/2018/G6P6	porcine	China	2018	ON068581

The RVC VP7 genes used in this study.

Strain	Host	Country	Collection date	Accession No.
RVC/Pig-wt/KOR/06-281-4/2006/G6Px	Porcine	South Korea	2004	KJ814496
RVC/Pig-wt/KOR/07-109-12/2007/G6Px	Porcine	South Korea	2004	KJ814498
RVC/Pig-wt/USA/OK-264/2015	Pig	USA	2015	MF522700
RV0143	Pig	USA	Dec-2011	MN809638
RVC/Pig-wt/USA/OK-179/2014	Pig	USA	2014	MF522615
RVC/pig-wt/USA/IL10-31/2010/G6Px	Pig	USA: Illinois	02-Mar-2010	JX273328
RVC/pig-wt/USA/IL10-33/2010/G6Px	Pig	USA: Illinois	02-Mar-2010	JX273330
RVC/Pig-wt/USA/OK-213/2014	Pig	USA	2014	MF522649
RVC/Pig-wt/USA/IA-136/2013	Pig	USA	2013	MF522572
RVC/pig-wt/USA/MN09-19/2009/G6Px	Pig	USA: Minnesota	23-Apr-2009	JX273316
RVC/pig-wt/USA/MN09-14/2009/G6Px	Pig	USA: Minnesota	23-Apr-2009	JX273311
RVC/SZ94/CHN/2011	Homo sapiens	China	2011	KP342029
RVC/SZ272/CHN/2011	Homo sapiens	China	2011	KP342040
RVC/SJZ217/CHN/2015	Homo sapiens	China	Apr-2015	KY865349
RVC/pig-wt/USA/AR09-13/2009/G1Px	Pig	USA: Arkansas	27-Mar-2009	JX273310
118-05-27	Pig	Italy	2007	EF464649
RVC/Pig-wt/USA/MN15-3/2012/G1P1	Pig	USA:MN	13-Feb-2012	MG451742
42/05-21	Pig	Italy	2007	EF464650
RVC/Pig-wt/KOR/11-58-7/2011/GxPx	Porcine	South Korea	2004	KJ814507
RVC/Pig-wt/KOR/09-84-5/2009/GxPx	Porcine	South Korea	2008	KJ814505
RVC/Pig-wt/USA/OK.5.68/2008	Sus scrofa	USA	02-Jan-2008	MH282893
RVC/Pig-wt/Tochigi-1-1/2015/G9P4	Sus scrofa	Japan: Tochigi	2015-11-27	LC122589
93-H5	Porcine	Japan:Gifu	2008-10	AB905227
RVC/Pig-wt/CZE/P59/2013	Pig	Czech Republic	Mar-2013	KM099271
RVC/Pig-wt/CZE/P303/2011	Pig	Czech Republic	Dec-2011	KM099268
RVC/pig-wt/USA/MN09-10/2009/G5Px	Pig	USA: Minnesota	18-Mar-2009	JX273307
CJ33-5	Sus scrofa	Japan:Aomori	2002-11	AB905216
134/04-18	Pig	Italy	2004	EF464653
RVC/Pig-wt/Tottori-KT01/2015/G13P4	Sus scrofa	Japan: Tottori	2015-07-15	LC122610
RVC/Pig-wt/CZE/P8/2011	Pig	Czech Republic	Nov-2010	KM099266
RVC/Pig-wt/KOR/2846/2012/G3Px	Porcine	South Korea	2004	KJ814494
Toyama	Bovine	Japan	2010	AB738417
Yamagata	Bovine	Japan	2010	AB108681
PoRVC_VP7_VIRES_GZ04_C1	Sus scrofa	China	Apr-2017	MK379227
PoRVC_VP7_VIRES_HeB02_C1	Sus scrofa	China	Apr-2017	MK379297
PoRVC_VP7_VIRES_NX01_C	Sus scrofa	China	Apr-2017	MK379437
RVC/CHN/JS02/2018/G6P6	Sus scrofa	China	2018	ON068582

The RVC NSP1 genes used in this study.

Strain	Host	Country	Collection date	Accession No.
PoRVC_NSP1_VIRES_HeB02_C2	Sus scrofa	China	Apr-2017	MK379284
PoRVC_NSP1_VIRES_NM02_C1	Sus scrofa	China	Apr-2017	MK379385
PoRVC_NSP1_VIRES_NM02_C2	Sus scrofa	China	Apr-2017	MK379386
CJ33-5	Sus scrofa	Japan:Aomori	2002-11	LC306977
Cowden				X60546
91-H5	Sus scrofa	Japan:Chiba	2008-10	LC306986
91-G7	Sus scrofa	Japan:Chiba	2008-10	LC306984
91-G10	Sus scrofa	Japan:Chiba	2008-10	LC306985
CJ16-4	Sus scrofa	Japan:Ehime	2002-06	LC306973
CJ59-32	Sus scrofa	Japan:Iwate	2003-10	LC306979
PoRVC_NSP1_VIRES_NM01_C3	Sus scrofa	China	Apr-2017	MK379353
RVC/Pig-wt/Tottori-KT01/2015/G13P4	Sus scrofa	Japan: Tottori	2015-07-15	LC122616
CJ32-3	Sus scrofa	Japan:Akita	2002-10	LC306976
RVC/Pig-wt/Tochigi-2/2014/G6P4	Sus scrofa	Japan: Tochigi	2014-04-25	LC122605
RVC/Pig-wt/Ishi-1/2015/G13P4	Pig	Japan: Ishikawa	2015-10-23	LC122627
CJ49-4	Sus scrofa	Japan:Aomori	2003-08	LC306978
RVC/Pig-wt/Tochigi-1-1/2015/G9P4	Pig	Japan: Tochigi	2015-11-27	LC122595
CJ10-1	Sus scrofa	Japan:Kagoshima	2002-05	LC306971
RVC/Dog-wt/HUN/174/2012/G10P8	dog	Hungary	2012	KP988019
Y/1/04	Bos taurus	Japan:Yamagata	2004-01-05	AB874643
Y/03	Bos taurus	Japan:Yamagata	2003-12-17	AB874642
Shintoku	Bos taurus	Japan		AB738402
Toyama	Bos taurus	Japan		AB738407
BK0830	Homo sapiens	Japan	2008	HQ185679
Bristol	Homo sapiens	United Kingdom		AJ132204
V508	Homo sapiens	India		AY770977
CAU 10-312	Homo sapiens	South Korea	2010	HQ896716
134-9	Sus scrofa	Japan:Miyazaki	2010-03	LC306990
CJ27-1	Sus scrofa	Japan:Aomori	2002-09	LC306974
105-4	Sus scrofa	Japan:Gifu	2009-03	LC306989
93-Z4	Sus scrofa	Japan:Gifu	2008-10	LC306988
93-H5	Sus scrofa	Japan:Gifu	2008-10	LC306987
RVC/CHN/JS02/2018/G6P6	Sus scrofa	China	2008	ON068572

The RVC NSP2 genes used in this study.

Strain	Host	Country	Collection date	Accession No.
PoRVC_NSP2_VIRES_HeB02_C1	Sus scrofa	China	Apr-2017	MK379286
RVC/Pig-wt/CZE/P141/2010	Sus scrofa	Czech Republic	2010	KP776736
Cowden				X65939
RVC/Pig-wt/CZE/P21/2013	pig	Czech Republic	Mar-2013	KP776735
RVC/Pig-wt/Ishi-1/2015/G13P4	Sus scrofa	Japan: Ishikawa	2015-10-23	LC122628
93-Z4	Sus scrofa	Japan:Gifu	2008-10	LC307009
CJ49-4	Sus scrofa	Japan:Aomori	2003-08	LC306999
CJ16-4	Sus scrofa	Japan:Ehime	2002-06	LC306994
93-H5	Sus scrofa	Japan:Gifu	2008-10	LC307010
CJ31-6	Sus scrofa	Japan:Kagoshima	2002-10	LC306996
91-G7	Sus scrofa	Japan:Chiba	2008-10	LC307007
91-H5	Sus scrofa	Japan:Chiba	2008-10	LC307006
CJ33-5	Sus scrofa	Japan:Aomori	2002-11	LC306998
BS347	Homo sapiens	Bangladesh	2005	HQ185639
V508	Homo sapiens	India		AY770979
Bristol	Homo sapiens	United Kingdom		AJ132205
YNR001	Homo sapiens	China	2007	HQ185659
Y/1/04	Bos taurus	Japan:Yamagata	2004-01-05	AB874649
Toyama	Bos taurus	Japan		AB738408
Shintoku	Bos taurus	Japan		AB738403
Y/3/04	Bos taurus	Japan:Yamagata	2004-06-02	AB874651
RVC/Dog-wt/HUN/174/2012/G10P8	Dog	Hungary	2012	KP988020
RVC/Pig-wt/Tottori-KT01/2015/G13P4	Sus scrofa	Japan: Tottori	2015-07-1	LC122617
CJ13-6	Sus scrofa	Japan:Kagoshima	2002-05	LC306993
CJ10-1	Sus scrofa	Japan:Kagoshima	2002-05	LC306992
87-I4	Sus scrofa	Japan:Ibaraki	2008-09	LC307004
134-9	Sus scrofa	Japan:Miyazaki	2010-03	LC307012
RVC/CHN/JS02/2018/G6P6	Sus scrofa	China	2018	ON068573

The RVC NSP3 genes used in this study.

Strain	Host	Country	Collection date	Accession No.
RVC/Pig-wt/Tochigi-1-1/2015/G9P4	Sus scrofa	Japan: Tochigi	2015-11-27	LC122597
87-I4	Sus scrofa	Japan:Ibaraki	2008-09	LC307027
CJ32-3	Sus scrofa	Japan:Akita	2002-10	LC307019
93-Z4	Sus scrofa	Japan:Gifu	2008-10	LC307031
105-4	Sus scrofa	Japan:Gifu	2009-03	LC307033
CAU13-1-77	Homo sapiens	South Korea	2013	KT355392
Bristol	Homo sapiens	United Kingdom	1999	AJ132203
RVC/SZ94/CHN/2011	Homo sapiens	China	2011	KP342032
Y/1/04	Bos taurus	Japan:Yamagata	2004-01-05	AB874655
Wu82	Homo sapiens	China	2001	HQ185649
Y/10	Bos taurus	Japan:Yamagata	2010-11-04	AB874659
RVC/Pig-wt/CAN/A8-158/2014/G6P4	pig	Canada	2014	KY909964
Y/08	Bos taurus	Japan:Yamagata	2008-04-08	AB874658
RVC/Pig-wt/Ishi-1/2015/G13P4	Sus scrofa	Japan: Ishikawa	2015-10-23	LC122629
VC/Pig-wt/Tochigi-2/2014/G6P4	Sus scrofa	Japan: Tochigi	2014-04-25	LC122607
CJ13-6	Sus scrofa	Japan:Kagoshima	2002-05	LC307015
RVC/Dog-wt/HUN/174/2012/G10P8	dog	Hungary	2012	KP988021
CJ10-1	Sus scrofa	Japan:Kagoshima	2002-05	LC307014
87-G2	Sus scrofa	Japan:Ibaraki	2008-09	LC307026
CJ3-6	Sus scrofa	Japan:Kagoshima	2002-05	LC307013
CJ16-4	Sus scrofa	Japan:Ehime	2002-06	LC307016
CJ31-6	Sus scrofa	Japan:Kagoshima	2002-10	LC307018
93-H5	Sus scrofa	Japan:Gifu	2008-10	LC307032
CJ33-5	Sus scrofa	Japan:Aomori	2002-11	LC307020
RVC/Pig-wt/Tottori-KT01/2015/G13P4	Sus scrofa	Japan: Tottori	2015-07-15	LC122618
PoRVC_NSP3_VIRES_GZ04_C1	Sus scrofa	China	Apr-2017	MK379219
134-9	Sus scrofa	Japan:Miyazaki	2010-03	LC307034
Cowden				M69115
RVC/CHN/JS02/2018/G6P6	Sus scrofa	China	2018	ON068574

The RVC NSP4 genes used in this study.

Strain	Host	Country	Collection date	Accession No.
RVC/Pig-wt/IND/Por-993/2015	porcine	India	2015	KY783644
87-I4	Sus scrofa	Japan:Ibaraki	2008-09	LC307049
CJ32-3	porcine	Japan:Akita	2002-10	LC307041
RVC/Pig-wt/CZE/P8/2011	pig	Czech Republic	Nov-2010	KM099248
RVC/Pig-wt/CZE/P9/2011	pig	Czech Republic	Nov-2010	KM099249
91-G10	pig	Japan:Chiba	2008-10	LC307050
RVC/Pig-wt/Tottori-KT01/2015/G13P4	pig	Japan: Tottori	2015-07-15	LC122619
105-4	Sus scrofa	Japan:Gifu	2009-03	LC307055
93-Z4	Sus scrofa	Japan:Gifu	2008-10	LC307054
93-H5	pig	Japan:Gifu	2008-10	LC307053
CJ59-32	pig	Japan:Iwate	2003-10	LC307044
RVC/Pig-wt/Tochigi-1-1/2015/G9P4	Sus scrofa	Japan: Tochigi	2015-11-27	LC122598
CAU 10-312	Homo sapiens	South Korea	2010	HQ896719
BS347	Homo sapiens	Bangladesh	2005	HQ185641
Bristol	Homo sapiens			X83967
YNR001	Homo sapiens	China	2007	HQ185661
Y/1/04	Bos taurus	Japan:Yamagata	2004-01-05	AB874661
Toyama	Bos taurus	Japan		AB738410
Y/10	Bos taurus	Japan:Yamagata	2010-11-04	AB874665
Shintoku	Bos taurus	Japan		AB738404
RVC/Dog-wt/HUN/174/2012/G10P8	dog	Hungary	2012	KP988022
RVC/CHN/JS02/2018/G6P6	Sus scrofa	China	2018	ON068575

The RVC NSP5 genes used in this study.

Strain	Host	Country	Collection date	Accession No.
RVC/Pig-wt/CZE/P8/2011	pig	Czech Republic	Nov-2010	KP776737
RVC/Pig-wt/CZE/P9/2011	pig	Czech Republic	Nov-2010	KP776738
RVC/Pig-wt/CZE/P21/2013	pig	Czech Republic	Mar-2013	KP776739
RVC/Pig-wt/CZE/P44/2013	pig	Czech Republic	Mar-2013	KP776740
RVC/Pig-wt/CZE/P59/2013	pig	Czech Republic	Mar-2013	KP776741
RVC/Pig-wt/CZE/P141/2010	pig	Czech Republic	Sep-2010	KP776742
RVC/Pig-wt/CZE/P303/2011	pig	Czech Republic	Dec-2011	KP776743
RVC/Pig-wt/Tochigi-1-1/2015/G9P4	Sus scrofa	Japan: Tochigi	2015-11-27	LC122599
RVC/Pig-wt/Tochigi-2/2014/G6P4	Sus scrofa	Japan: Tochigi	2014-04-25	LC122609
RVC/Pig-wt/Tottori-KT01/2015/G13P4	Sus scrofa	Japan: Tochigi	2015-07-15	LC122620
RVC/Pig-wt/Ishi-1/2015/G13P4	Sus scrofa	Japan: Tochigi	2015-10-23	LC122631
Cowden	Sus scrofa	USA		X65938
CAU 10-312	Homo sapiens	South Korea	2010	HQ896720
BS347	Homo sapiens	Bangladesh	2005	HQ185642
V508	Homo sapiens	India		AY770978
YNR001	Homo sapiens	China	2007	HQ185662
Y/1/04	Bos taurus	Japan:Yamagata	2004-01-05	AB874667
Y/08	Bos taurus	Japan:Yamagata	2008-04-08	AB874670
Y/3/04	Bos taurus	Japan:Yamagata	2004-06-02	AB874669
Toyama	Bos taurus	Japan		AB738411
RVC/Dog-wt/HUN/174/2012/G10P8	Dog	Hungary	2012	KP988023
RVC/CHN/JS02/2018/G6P6	Sus scrofa	China	2018	ON068576
