# Supplementary figures and images for: Genome Analysis of the G6P6 Genotype of Porcine Group C Rotavirus in China
Source: Animals (Basel). 2022 Oct 27;12(21):2951. doi: 10.3390/ani12212951 (PMC9657714; doi:10.3390/ani12212951)

Figure S1

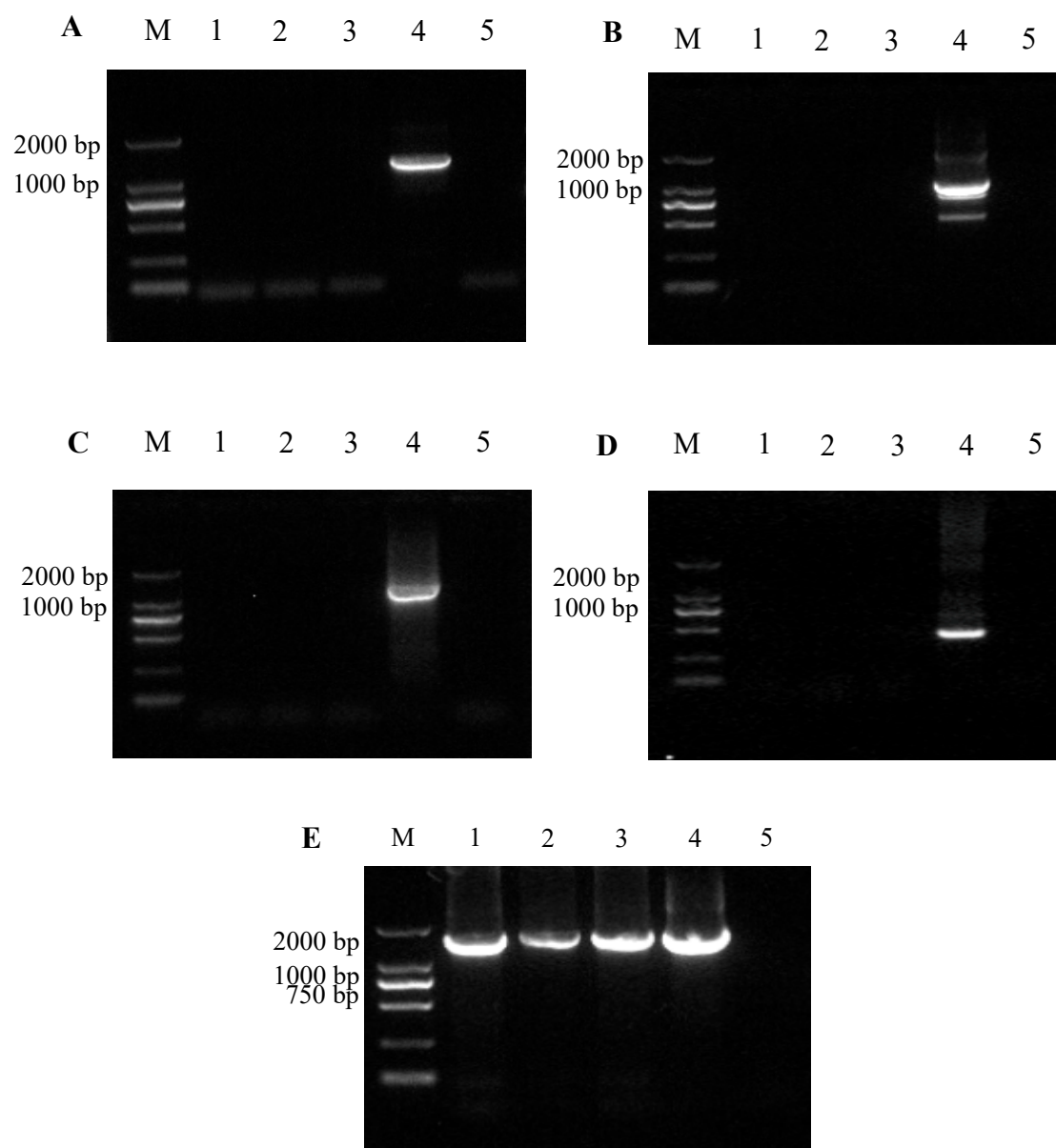

Supplement: Supplementary file 1 [file animals-12-02951-s001.zip › animals-1997300-Figure S1.pdf]

Figure S2

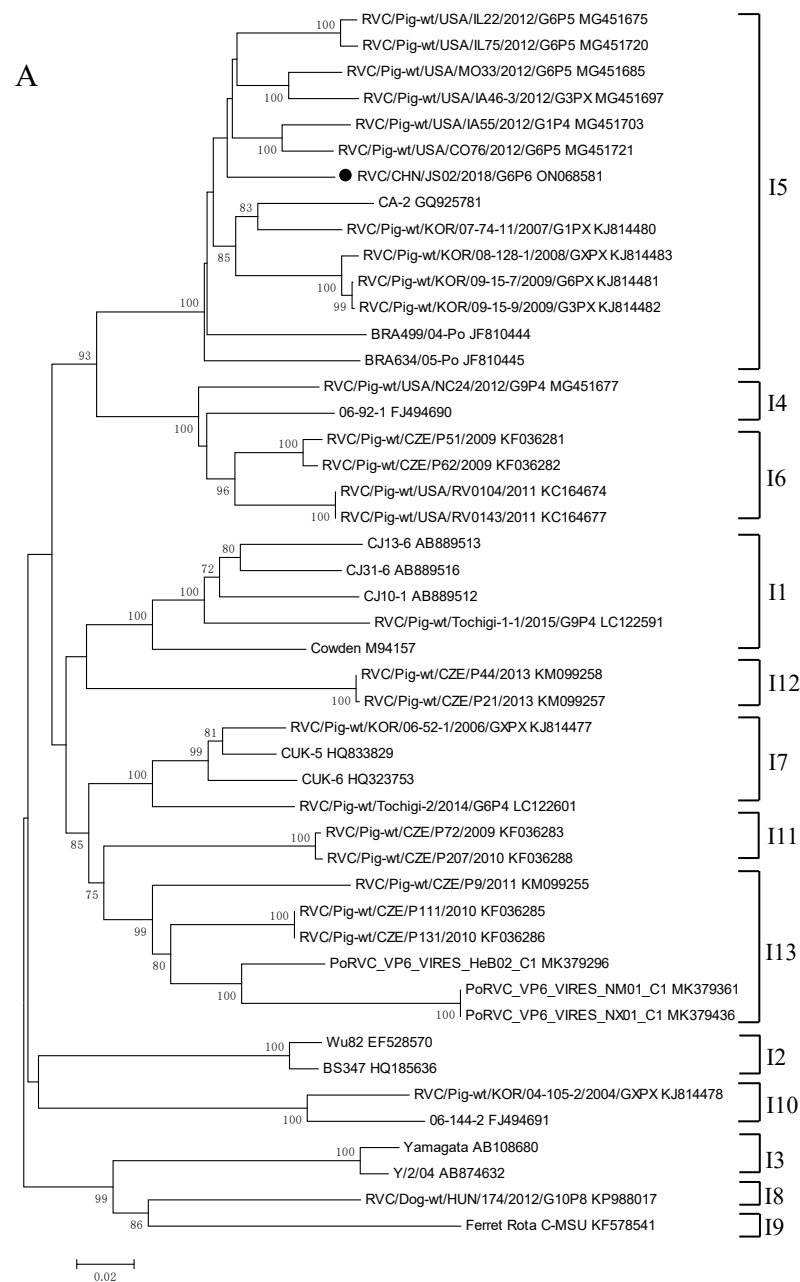

Figure S2 continue

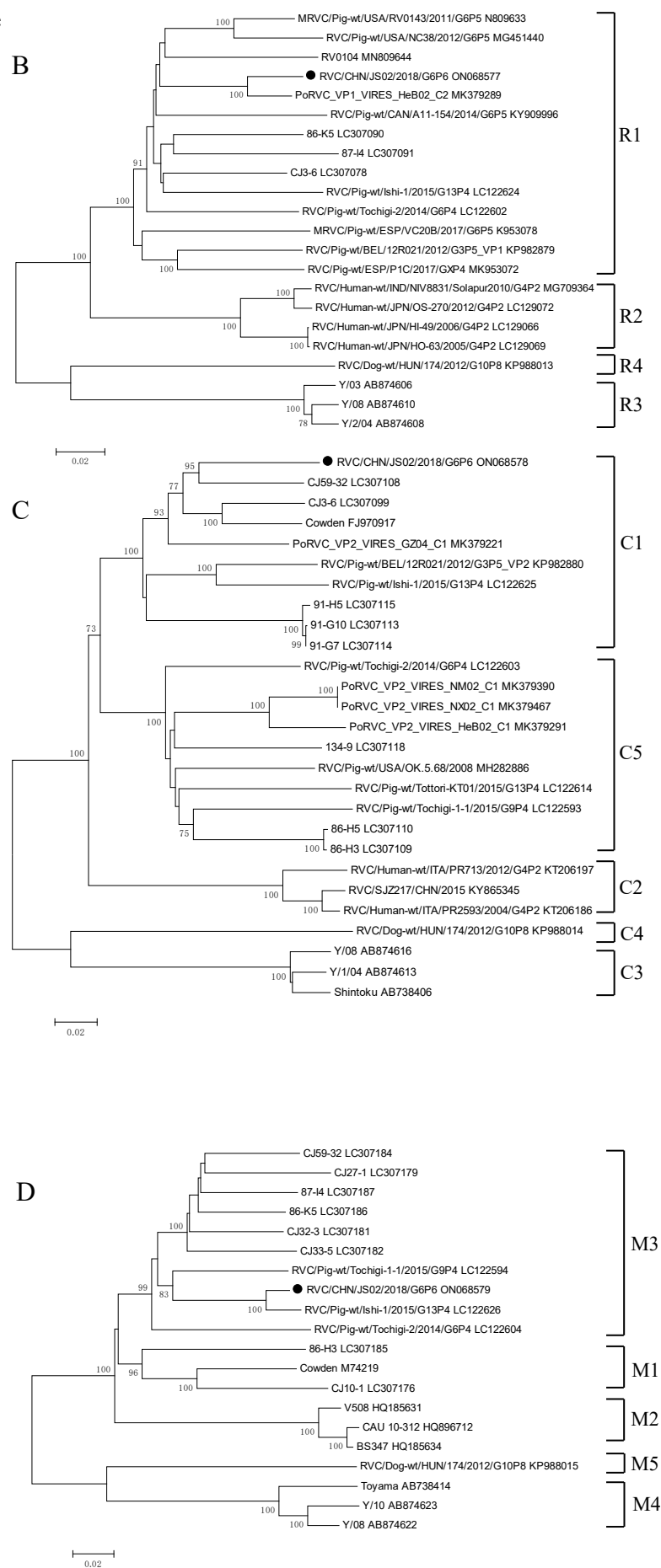

Figure S2 continue

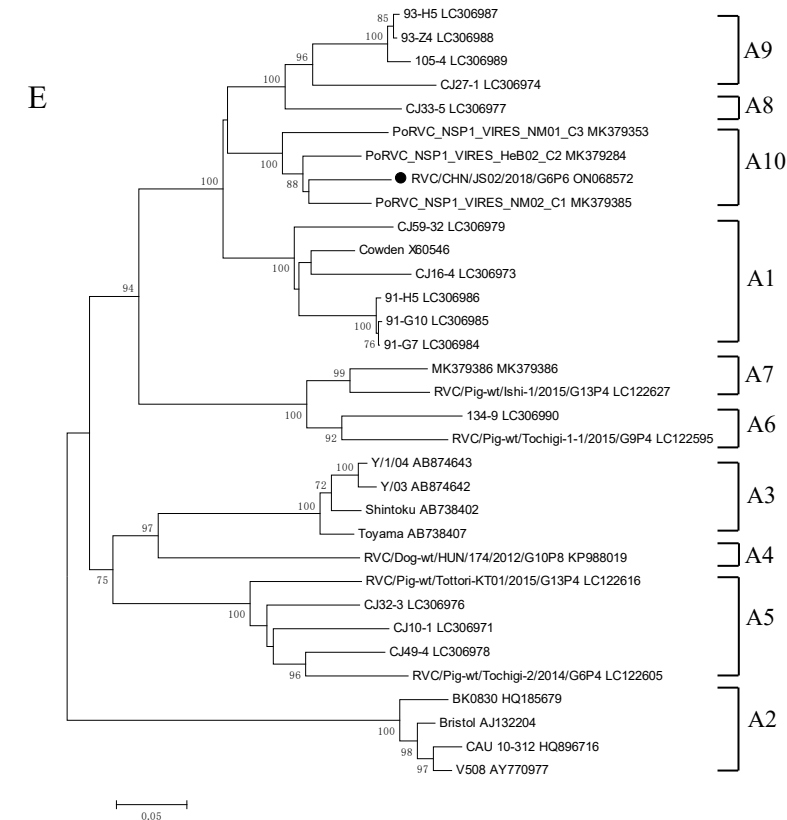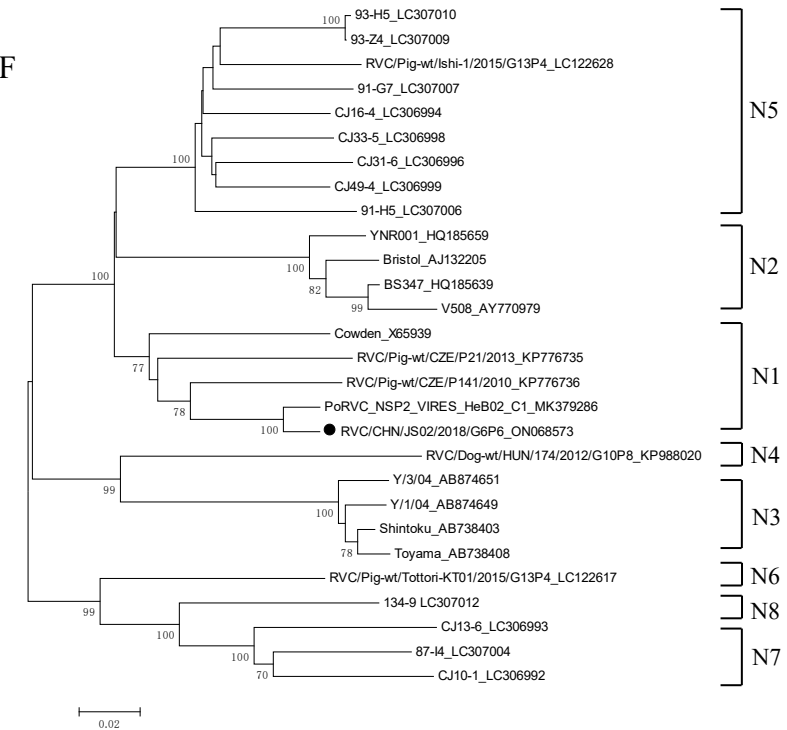

Figure S2 continue

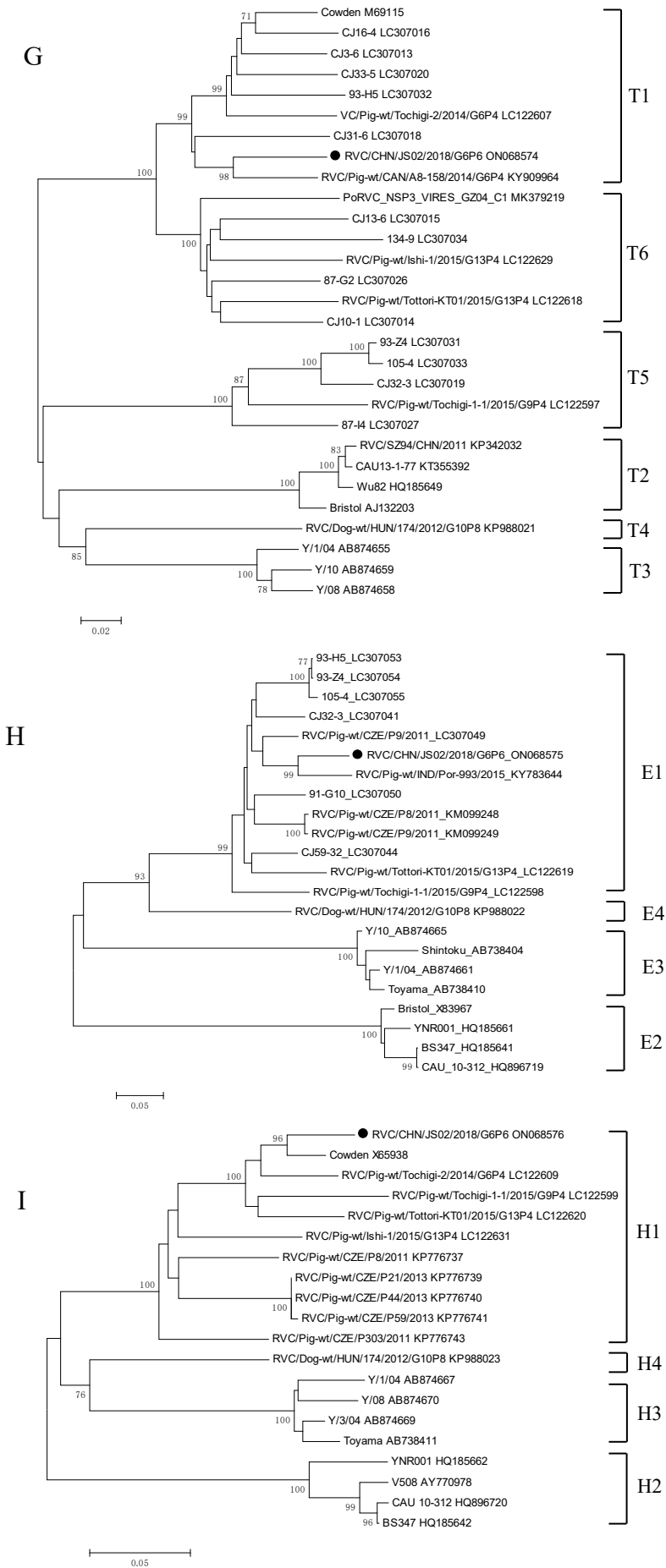

Supplement: Supplementary file 1 [file animals-12-02951-s001.zip › animals-1997300-Figure S2.pdf]
